# Nephrogenic diabetes insipidus after esophagectomy in a patient with remote history of lithium treatment: A case report

**DOI:** 10.1016/j.ijscr.2019.03.006

**Published:** 2019-03-19

**Authors:** Dania Shakaroun, Hassan Nasser, Semeret Munie, Sandeep Soman

**Affiliations:** a Department of Internal Medicine, Henry Ford Hospital, Detroit, MI, USA; b Department of Surgery, Henry Ford Hospital, Detroit, MI, USA; c Department of Surgery, Division of Trauma and Critical Care, Henry Ford Hospital, Detroit, MI, USA; d Department of Internal Medicine, Division of Nephrology and Hypertension, Henry Ford Hospital, Detroit, MI, USA

**Keywords:** NDI, nephrogenic diabetes insipidus, ADH, anti-diuretic hormone, NPO, nil per os, D5W, dextrose 5% in water, cAMP, cyclic adenosine monophosphate, Case report, Lithium, Nephrogenic diabetes insipidus, Hypernatremia, Esophagectomy, Esophageal cancer

## Abstract

**Introduction:**

Nephrogenic diabetes insipidus occurs in patients on chronic lithium treatment even after lithium discontinuation. Patients affected by this disorder are highly vulnerable to hypernatremia when they cannot respond to their thirst mechanism. We report a rare case of hypernatremia due to undiagnosed nephrogenic diabetes insipidus post esophagectomy in a patient with remote history of lithium use.

**Presentation of case:**

A 70-year-old female with past medical history of bipolar disorder, chronic kidney disease and pheochromocytoma underwent an elective esophagectomy for esophageal adenocarcinoma. Lithium was discontinued 10 years prior to her presentation. She was kept nil per os post operatively and subsequently developed altered mental status necessitating intubation. Her sodium level was found to be 156 mmol/L. A water deprivation test and desmopressin trial confirmed nephrogenic diabetes insipidus. Days after dextrose 5% in water infusion, free water flushes through the jejunostomy tube and hydrochlorothiazide, her hypernatremia improved slowly with subsequent improvement in her mental status.

**Discussion:**

Several mechanisms have been described in literature to explain the persistent damage caused by lithium on the kidneys. When patients lose access to a source of free water and are resuscitated with normal saline post operatively, they are at risk of developing life-threatening hypernatremia. This can be avoided by aggressive hydration with appropriate fluid replacement.

**Conclusion:**

Surgeons should be aware of the persistent renal defects caused by long term lithium use and development of nephrogenic diabetes insipidus even years after medication cessation.

## Highlights

• Nephrogenic diabetes insipidus can occur years after lithium discontinuation.
• Life-threatening hypernatremia can develop post-operatively.
• Inappropriate fluid resuscitation worsens post-operative hypernatremia.
• High clinical suspicion and close monitoring are essential in the post-operative period.

## 1 Introduction

Diabetes insipidus, which is clinically characterized by polyuria and polydipsia, is a disorder resulting from inadequate anti-diuretic hormone (ADH) action. Nephrogenic diabetes insipidus (NDI) occurs when there is normal ADH secretion but abnormal renal response to its diuretic effect causing inability to concentrate urine. NDI can be either hereditary or acquired due to hypercalcemia, obstructive uropathy, hypokalemia or drugs. Chronic lithium use is considered one of the most common causes of NDI. It is estimated that 12% of patients on chronic lithium treatment develop NDI [1]. Even after lithium discontinuation, some patients are still at risk of developing NDI due to persistent renal concentrating defect [2]. This risk can be easily missed if physicians are unaware of the remote history of lithium use. NDI can manifest as a serious postoperative complication if fluid intake is restricted, and the inappropriate intravenous fluids are administered. It is crucial for surgeons to recognize NDI and initiate early therapy, as it can lead to serious neurologic consequences. We report a rare case of hypernatremia due to undiagnosed NDI post esophagectomy in a patient with remote history of lithium use. This case report has been reported in line with the surgical case report (SCARE) criteria [3].

## 2 Presentation of case

A 70-year-old female with past medical history of bipolar disorder, chronic kidney disease and pheochromocytoma status post adrenalectomy was admitted to the hospital for an elective esophagectomy for esophageal adenocarcinoma. Her bipolar disorder was previously treated with lithium for 25 years but was discontinued 10 years prior to her presentation.

The patient underwent robotic-assisted laparoscopic esophagectomy and jejunostomy feeding tube insertion with no significant complications. Post-operatively, she was kept nil per os (NPO) and started on 0.9% sodium chloride infusion at a rate of 75 ml/hour. She subsequently developed confusion, agitation, anxiety and tremulousness. She pulled out her nasogastric tube and attempted to pull out her peripheral intravenous lines. Blood work revealed an increase in her serum sodium from 145 to 156 mmol/L and creatinine from 1.57 to 1.98 mmol/L within 24 h with normal potassium level.

Intravenous 0.9% saline infusion was replaced by dextrose 5% in water (D5W) infusion along with free water through the jejunostomy tube with no significant improvement in her sodium level. On post-operative day 3, the patient’s clinical status deteriorated. She had significant mental status changes and was found unresponsive necessitating intubation for airway protection. She was subsequently transferred to the surgical intensive care unit. Follow-up labs showed persistently elevated sodium level at 155 mmol/L, urine osmolarity of 299 mOsm/kg H_2_O and serum osmolarity of 334 mOsm/kg H_2_O (Table 1). She had a urine output of 4.2 L in 24 h. A ‘water deprivation test’ was done, the results of which were consistent with diabetes insipidus. On post-operative day 5, a trial dose of 4 μg of intravenous desmopressin was administered with no significant increase in urine osmolarity consistent with NDI (Table 1). She was subsequently started on hydrochlorothiazide 25 mg daily.

**Table 1:** Patient's lab values and urine output postoperatively.

POD	0	1	2	3	4	5	6	7	8	9
S_Na_	141	145	156	155	156	153	152	150	142	140
S_Osm_				334		321			301	
U_Osm_				299	209	219	211	224	192	
U_Na_				85	51	65	76	72	42	
UO	3410	2425	2700	4255	4080	3705	4000	2050	1350	1625

POD, postoperative day; S_Na_, serum sodium in mmol/L; S_Osm_, serum osmolarity in mOsm/kg H_2_O; U_Osm_, urine osmolarity in mOsm/kg H_2_O; U_Na_, urine sodium in mmol/L; UO, urine output in mL per day.

After a few days of free water flushes through the jejunostomy feeding tube, D5W infusion and hydrochlorothiazide, the patient’s sodium level started to improve slowly reaching 140 mmol/L on post-operative day 9. D5W infusion was subsequently stopped and free water flushes were adjusted to 300 mL every 6 h. The patient’s mental status improved gradually as her sodium level normalized. She was extubated and transferred to a surgical step-down unit. She was discharged on post-operative day 18. She was seen in clinic 4 months later where she denied polydipsia and polyuria and her sodium level was 141 mmol/L.

## 3 Discussion

Lithium is one of the main maintenance treatment options for bipolar disorder. It is used in up to 0.1% of the entire population [2]. It is widely used despite its various renal side effects that include chronic tubulointerstitial nephropathy, renal tubular acidosis, hypercalcemia and NDI.

ADH or vasopressin binds to its V2 receptors on the principal cells of the collecting tubules leading to aquaporin-2 water channels translocation and water reabsorption down a favorable concentration gradient. Behl et al. described multiple mechanisms by which lithium causes natriuresis and renal cellular damage including interference with epithelial sodium channels and increase in cyclooxygenase-2 and prostaglandin E2 expression in the cortical collecting tubules [4,5]. These described changes explain the acute effect of lithium on the renal tubules but do not explain the persistent concentrating defect after withdrawal of the medication. Several mechanisms of persistent damage caused by lithium have been studied in literature and include slow recovery of aquaporin-2 gene expression after lithium cessation, loss of renal medullary osmotic gradient and lithium-induced interstitial nephritis that causes persistent renal insufficiency [6]. To note, our patient did have a history of chronic kidney disease that was attributed to lithium and could have contributed to her persistent renal concentrating defect 10 years after stopping the medication.

Patients with lithium-induced NDI usually report a history of polydipsia and polyuria and do not develop hypernatremia as long as they are able to keep up with their renal water losses. When these patients cannot respond to their thirst drive, they develop hypernatremia with increased serum osmolality and decreased urine osmolality because of the kidneys’ inability to concentrate urine and maintain appropriate fluid balance. This is what happened to our patient who was kept NPO after her surgery and was resuscitated with 0.9% sodium chloride which gave an additional sodium load that the kidneys were unable to eliminate. Interestingly, our patient underwent an adrenalectomy for a diagnosis of pheochromocytoma few months prior to this presentation but did not develop hypernatremia because she had access to free water post-operatively. Post-operative hypernatremia can be fatal. Baraza et al. reported a case of a large bowel obstruction from sigmoid adenocarcinoma that necessitated an urgent laparotomy with hemicolectomy [7]. The patient developed hypernatremia reaching 185 mmol/L due to lithium-induced NDI and suffered a respiratory arrest. Hypernatremia from lithium-induced NDI has also been reported in the literature after gastric banding and coronary artery bypass [6,8].

The diagnosis of lithium-induced NDI depends on a detailed history of current or remote lithium use, symptoms of polydipsia and polyuria and absence of other causes of NDI. Our patient had no evidence of hypokalemia, hypercalcemia, or medication use that might have led to NDI other than her remote lithium use. Diabetes insipidus diagnosis is suggested by high serum sodium concentration and low urine osmolality. A ‘water deprivation test’ usually confirms the diagnosis and patients’ inability to concentrate urine. The test is abnormal when urine osmolality does not increase after a period (usually 12 h) of depriving patients of water. Normal patients have more than 100% increase in their urine osmolality. To determine whether the diabetes insipidus is of nephrogenic or central origin, 4 μg of desmopressin subcutaneously or intravenously should be administered. A less than 10% increase in urine osmolality after desmopressin is consistent with NDI [2]. Hypo-osmolar solutions are used to correct the water deficit and replace ongoing water losses based on the calculated total body water deficits. Thiazide diuretics, amiloride and indomethacin are also used to correct hypernatremia. Diuretics promote proximal tubular water reabsorption, and hence decreased free water transmission to the distal collecting tubules where the urine concentrating defect is located. Indomethacin inhibits prostaglandin which increases cAMP in the collecting tubules and thus increase water reabsorption [2]. Unlike central diabetes insipidus, treatment with desmopressin fails to improve hypernatremia, serum osmolality and urine osmolality in most cases [9].

Hypernatremia due to lithium-induced NDI is a rare but potentially fatal post-operative complication. Surgeons should have a high index of suspicion when patients with history of lithium use develop hypernatremia and neurologic changes. A history of bipolar disorder should entail a more detailed inquiry about lithium use and symptoms of polyuria and polydipsia in the pre-operative evaluation. This allows preemptive measures to avoid the possibly detrimental complications of hypernatremia in the post-operative period. It is crucial in such patients to have more frequent electrolytes checks, more accurate urine output measures and closer monitoring of their neurologic status [6]. It is preferable that patients at high risk of developing of hypernatremia due to lithium-induced NDI be allowed access to free water as soon as it is clinically appropriate to replace their ongoing water losses. Hypo-osmolar solutions such as D5W or half normal saline should be the maintenance fluids of choice in the post-operative period instead of normal saline that worsens hypernatremia. An early consultation to nephrology service can be important, as other modalities can be used to correct the hypernatremia and reverse any neurologic changes.

## 4 Conclusion

Lithium is a common cause of nephrogenic diabetes insipidus. Lithium’s concentrating defect can persist years after its cessation. Hypernatremia due to NDI can be a major cause of morbidity and mortality in the post-operative period when patients lose access to free water. Surgeons need to be aware of lithium induced NDI and the potential rapid onset of life threatening neurologic and metabolic disorders. Worsening post-operative hypernatremia and polyuria showed prompt early consideration of lithium-induced NDI and aggressive rehydration with appropriate fluids to prevent detrimental neurologic changes.

## Conflicts of interest

No conflicts of interest to be declared.

## Sources of funding

No source to be stated.

## Ethical approval

No Institutional Review Board is required for publication of a case report at our institution.

## Consent

Written informed consent was obtained by the patient for publication of this case report and accompanying images. A copy of the written consent is available for review by the Editor-in-Chief of this journal on request.

## Author contribution

Dania Shakaroun: Formal analysis; Writing – original draft.

Hassan Nasser: Writing – original draft.

Semeret Munie: Writing – review & editing.

Amy Li: Writing – review & editing.

Sandeep Soman: Supervision; Writing – review & editing.

## Registration of research studies

Not applicable.

## Guarantor

Sandeep Soman, MD.

Provenance and peer review

Not commissioned, externally peer reviewed.
